# Are Current UK National Institute for Health and Clinical Excellence (NICE) Obesity Risk Guidelines Useful? Cross-Sectional Associations with Cardiovascular Disease Risk Factors in a Large, Representative English Population

**DOI:** 10.1371/journal.pone.0067764

**Published:** 2013-07-02

**Authors:** Faiza Tabassum, G. David Batty

**Affiliations:** 1 Department of Infection and Population Health, University College London, London, United Kingdom; 2 Department of Epidemiology and Public Health, University College, London, United Kingdom; Old Dominion University, United States of America

## Abstract

The National Institute for Health and Clinical Excellence (NICE) has recently released obesity guidelines for health risk. For the first time in the UK, we estimate the utility of these guidelines by relating them to the established cardiovascular disease (CVD) risk factors. Health Survey for England (HSE) 2006, a population-based cross-sectional study in England was used with a sample size of 7225 men and women aged ≥35 years (age range: 35–97 years). The following CVD risk factor outcomes were used: hypertension, diabetes, total and high density lipoprotein cholesterol, glycated haemoglobin, fibrinogen, C-reactive protein and Framingham risk score. Four NICE categories of obesity were created based on body mass index (BMI) and waist circumference (WC): *no risk* (up to normal BMI and low/high WC); *increased risk* (normal BMI & very high WC, or obese & low WC); *high risk* (overweight & very high WC, or obese & high WC); and *very high risk* (obese I & very high WC or obese II/III with any levels of WC. Men and women in the very high risk category had the highest odds ratios (OR) of having unfavourable CVD risk factors compared to those in the no risk category. For example, the OR of having hypertension for those in the very high risk category of the NICE obesity groupings was 2.57 (95% confidence interval 2.06 to 3.21) in men, and 2.15 (1.75 to 2.64) in women. Moreover, a dose-response association between the adiposity groups and most of the CVD risk factors was observed except total cholesterol in men and low HDL in women. Similar results were apparent when the Framingham risk score was the outcome of interest. In conclusion, the current NICE definitions of obesity show utility for a range of CVD risk factors and CVD risk in both men and women.

## Introduction

It is very well documented that the prevalence of overweight and obesity in England and other high-income countries is increasing at all ages: almost two-thirds of adults and a third of children are so classified [1]. A series of studies have established that obesity, typically indexed by body mass index (BMI), is associated with premature mortality [2], [3]–[6] elevated rates of cardiovascular disease (CVD) risk [7], [8], selected cancers [9], [10], [11], disability [12]–[14] and, potentially, mental health problems [15]–[17]. More recently, investigators have shown that waist circumference (WC), an indicator of visceral fat, is also associated with these health outcomes [18]–[25].

The National Institute for Health and Clinical Excellence (NICE), a UK agency established in 1999 to assist health care professionals in providing the best care based on current scientific evidence, has endorsed using combined measures of general and central adiposity in estimating the ‘health’ risks associated with overweight and obesity [26]. However, the utility of these recommendations has yet to be assessed. Accordingly, we examined the predictive utility of these guidelines by relating them to both CVD risk factors and a CVD risk score (the Framingham index) using data from the Health Survey of England, a large, representative sample of that country’s population.

## Methods

### Data

The Health Survey for England (HSE) comprises a series of annual surveys beginning in 1991. HSE is a repeat, cross-sectional survey of independent samples designed to ascertain the prevalence of chronic diseases and their risk factors. Each year, a new, representative sample of the population living in private households is selected. Herein, we utilised data from the 2006 survey as this focused on cardiovascular disease risk factors. In the multi-stage stratified sampling process, 13,680 addresses were randomly identified. Up to 10 resident adults (aged 16 and over) at each selected private household address were eligible for inclusion in the survey. Interviews were held in 8,614 households with 14,142 adults aged 16 or over, of which 10,489 adults had a nurse visit [27]. Full details of sampling method can be found elsewhere [27]. Nurses obtained written consent from adults before taking blood samples. This study is an analysis of previously collected data and therefore ethical approval was not required for this study. Ethical approval for this survey was obtained by the Health Survey for England team from the London Multi-centre Research Ethics Committee.

### Assessment of Body Mass Index and Waist Circumference

At the interview stage, participants had their height measured using a portable stadiometer. Measurement was taken without shoes, with the participant stretching to their maximum height and the head positioned in the Frankfort plane. Readings were taken to the nearest millimetre. BMI was calculated using the usual formulae: weight in kilograms divided by the square of height in meters (kg/m^2^). The waist measurement was taken at the midpoint between the lower rib and the upper margin of the iliac crest. Measured using a tape with an insertion buckle at one end, two readings were taken with the mean used in the present analysis. Data from those who were considered by the interviewer to have unreliable measurements, for example those who had excessive clothing on, or women who were pregnant were excluded from the analysis. We used the combined categories of BMI and WC as defined by the NICE [26] which are based on three thresholds for WC and five for BMI (table 1). For the current analyses, we used the following four risk categories: *no risk, increased risk, high risk and very high risk*.

**Table 1:** NICE obesity categories based on combined BMI and WC.

BMI classification	Waist circumference*		
	Low	High	Very high
Normal weight (up to 30 kg/m^2^)	No increased risk	No increased risk	Increased risk
Overweight (25 to less than 30 kg/m^2^)	No increased risk	Increased risk	High risk
Obesity I (30 to less than 35 kg/m^2^)	Increased risk	High risk	Very high risk
Obesity II (35 to less than 40 kg/m^2^)	Very high risk	Very high risk	Very high risk
Obesity III (40 kg/m^2^ or more)	Very high risk	Very high risk	Very high risk

BMI: body mass index.

* Waist circumference is defined as: For men: low (<94 cm); high (94–102 cm); very high (>102 cm); For women: low (<80 cm); high (80–88 cm) and very high (>88 cm).

### CVD Risk Factors

A (non-fasting) sample of blood was taken by venepuncture from study members aged 16 and over. The blood sample was analysed for total and HDL cholesterol, glycated haemoglobin, fibrinogen, and C-reactive protein. The following risk categories were used for low HDL (<1.0 mmol/l in men, <1.3 mmol/l in women), hypertension (systolic blood pressure: ≥140 mmHg, diastolic blood pressure: ≥90 mmHg), and total cholesterol (≥5.0 mg/dL). The Framingham risk score was calculated based on the values of total cholesterol, HDL cholesterol, systolic and diastolic blood pressure, diabetes, age, gender and smoking status [28].

### Covariates

Participants were asked about their smoking behaviour during the face to face interview and categorised as: never smokers, ex-smokers and current smokers. The physical activity levels were assessed by questions on occupational activity, walking, and sport and recreation. Participants were asked about frequency, duration, and intensity of the different types of activity which last for at least 15 minutes in the 4 weeks prior to interview. They were classified as high (30 min or more of moderate intensity activity at least five days a week), medium (30 min or more of moderate intensity activity at least on 1 to 4 days a week), or low (lower levels of activity) [27]. Alcohol consumption was reported in terms of units of alcohol consumed on the heaviest drinking day in the past week; one unit of alcohol corresponds to 10 ml by volume of pure alcohol. Alcohol consumption was categorised into four groups: none; low (up to and including four units); medium (≥5 units but ≤8 units) and high (>8 units). Respondents were assigned occupational categories according to the National Statistics Socioeconomic Classification (NS-SEC) [29] on the basis of their most recent occupation. The following three categories of NS-SEC were used: managerial and professional, intermediate, and routine and manual occupations.

### Statistical Analysis

In the present analyses we utilise data from men and women aged 35 years or older. A total of 7225 individuals had data on BMI and WC, while the analytical sample varied according to the outcome under consideration (range: 4079 for complete data for the Framingham risk score to 7225 for HDL). Data were weighted for nonresponse to make the sample representative of the general population; when analysing blood sample data, weights were further corrected for nonresponse to the blood samples to reduce bias and produce results that remained nationally representative. In preliminary analyses, there was evidence that sex modified the relation of NICE categories with BMI and WC (*p*<0.05) with the biomarkers; as such, we present gender-specific results. Normality of biomarkers was assessed with CRP log transformed; geometric means are presented, and the natural log of the concentrations was used in the regression models. The relationship between each of the continuous outcomes and NICE was explored using ANOVA; for dichotomous outcomes, chi-squared test was used. Unadjusted and adjusted associations of NICE obesity categories with each of the outcome variables were estimated by using simple and multiple regression analysis. Logistic regression analyses were used when the outcome was categorical (for example hypertension: yes or no); linear regression when it was continuous (such as CRP). In multivariable analyses we controlled for smoking status, physical activity, alcohol consumption and occupational class. Analyses were carried out using STATA (version 11.0 for Windows; Stata Corporation, College Station, TX).

## Results

The characteristics of participants with respect to the NICE categories of adiposity are shown separately in men (table 2) and women (table 3). Participants in the low risk category were younger and had more favourable levels of CVD risk factors than those with high or very high risk category. For example, in the no risk category compared to those in the very high risk category, the levels of systolic blood pressure were 7 mm/Hg and 9 mm/Hg lower in men and women, respectively. HDL levels were also raised in the higher risk groups; the pattern of association for total cholesterol was less clear. The concentration levels of fibrinogen and CRP were significantly higher among those in very high obesity risk category compared to those in no risk category. The Framingham risk score was also highest among those with very high risk category (p-value for trend <0.001). In general, there was a graded linear relationship between risk categories of NICE and CVD risk factors such that the beta coefficients or the odds ratios of CVD risk factors increased in a step-wise manner with the increase in the risk category.

**Table 2:** Characteristics of participants (mean [sd]) by NICE obesity categories in the Health Survey of England (2006) – men.

	n	NICE adiposity categories				p-value for trend
		No risk	Increased risk	High risk	Very high risk	
Age (years)	3344	54.14 (14.13)	56.54 (13.26)	59.39 (13.33)	56.62 (12.53)	<0.001
BMI kg/m^2^	3344	24.15 (2.12)	27.23 (1.33)	28.67 (1.51)	33.51 (3.14)	<0.001
WC (cm)	3344	88.94 (5.56)	98.11 (2.44)	105.07 (4.10)	113.88 (8.37)	<0.001
Systolic blood pressure (mm/Hg)	2836	129.24 (15.30)	133.49 (16.27)	134.84 (15.72)	136.99 (16.95)	<0.001
Diastolic blood pressure (mm/Hg)	2836	73.51 (10.24)	75.62 (10.54)	76.10 (10.84)	78.29 (11.87)	<0.001
Total Cholesterol (mg/dL)	2526	5.44 (1.10)	5.63 (1.17)	5.52 (1.16)	5.49 (1.14)	0.50
HDL cholesterol (mg/dL)	2526	1.46 (0.37)	1.34 (0.32)	1.31 (0.32)	1.23 (0.29)	<0.001
HbA1C (mmol/l)	2499	5.49 (0.56)	5.61 (0.75)	5.80 (1.01)	5.88 (1.02)	<0.001
Framingham risk score*	1860	5.13 (3.10)	6.30 (2.93)	6.78 (2.94)	6.92 (2.66)	<0.001
Inflammatory markers						
Fibrinogen (g/L)	1952	2.86 (0.71)	2.96 (0.70)	3.03 (0.69)	3.13 (0.78)	<0.001
CRP (mg/L)	2526	1.10 (3.25)	1.60 (3.00)	2.06 (2.92)	2.44 (2.71)	<0.001
Prevalence (%)						
Hypertension	2836	22	32	35	40	<0.001
Type 2 diabetes	2872	3	5	7	13	<0.001
High cholesterol	2526	65	72	68	66	0.04
Low HDL levels	2526	4	7	11	13	<0.001

BMI: body mass index; WC: waist circumference; HDL: high density lipoprotein; HbA1C: glycated haemoglobin; CRP: C-reactive protein.

* Lower scores denoted lower risk of CVD.

**Table 3:** Characteristics of participants (mean [sd]) by NICE obesity categories in the Health Survey of England (2006) – women.

	n	NICE adiposity categories				p-value for trend
		No risk	Increased risk	High risk	Very high risk	
Age (years)	3881	52.89 (13.31)	55.92 (13.71)	59.25 (13.88)	56.83 (13.38)	<0.001
BMI kg/m^2^	3881	22.83 (2.08)	26.43 (1.67)	28.00 (1.47)	34.72 (4.11)	<0.001
WC (cm)	3881	76.69 (5.31)	85.39 (3.87)	93.59 (4.96)	104 17 (9.79)	<0.001
Systolic BP (mm/Hg)	3375	123.83 (18.68)	127.26 (18.68)	132.05 (20.47)	132.27 (18.25)	<0.001
Diastolic BP (mm/Hg)	3375	71.93 (10.22)	73.66 (10.27)	74.77 (10.66)	77.38 (10.93)	<0.001
Total Cholesterol (mg/dL)	2934	5.56 (1.06)	5.71 (1.11)	5.83 (1.24)	5.81 (1.19)	<0.001
HDL cholesterol (mg/dL)	2934	1.78 (0.42)	1.67 (0.36)	1.56 (0.35)	1.49 (0.32)	<0.001
HbA1C (mmol/l)	2906	5.43 (0.49)	5.48 (0.44)	5.65 (0.65)	5.80 (0.86)	<0.001
Framingham risk score*	2219	1.61 (4.17)	2.83 (4.16)	4.53 (4.32)	4.83 (4.23)	<0.001
Fibrinogen (g/L)	2337	2.91 (0.72)	3.08 (0.62)	3.16 (0.68)	3.39 (0.69)	<0.001
CRP† (mg/L)	2931	0.97 (3.26)	1.51 (2.87)	1.94 (2.76)	3.56 (2.56)	<0.001
Prevalence %						
Hypertension	3375	19	23	31	33	<0.001
Type 2 diabetes	3354	2	2	6	9	<0.001
High cholesterol	2934	70	74	76	76	0.009
Low HDL levels	2934	1.11	0.80	1.48	2.37	0.078

BMI: body mass index; WC: waist circumference; HDL: high density lipoprotein; HbA1C: glycated haemoglobin; CRP: C-reactive protein.

* Lower scores denoted lower risk of CVD.

† Geometric means are reported.

Next, we present regression models for the association of the NICE adiposity groupings with CVD risk factors. Unadjusted and adjusted regression analyses of CVD risk factors in relation to the adiposity categories of NICE are presented in Table 4. In both men and women, for hypertension, the odds ratios were higher across the three risk categories compared to no risk both. For example, the odds ratios were 2.57 (95% confidence interval 2.06 to 3.21) of hypertension for those in very high risk category compared to men in no risk category. A dose-response was also observed across the three categories such that odds ratios of hypertension increased with the increase in risk category (OR: 1.69, 1.95, 2.57 respectively for increased, high and very high risk categories). The odds ratios remained unchanged when covariates were added to the multivariable model. Likewise, HDL levels were lowest in very high risk category among men both in unadjusted and adjusted associations. The odds ratios were almost 6 times higher (95% confidence interval 3.59 to 8.76) for diabetes for those in very high risk category compared to no risk. Similarly, the levels of fibrinogen and CRP increased with the increase in the risk category and were highest for those in ‘very high risk category’. The total cholesterol levels were not associated with risk categories in men.

**Table 4:** Odds ratios or β coefficients (95% CI) for the relation of NICE obesity categories with CVD risk factors in the Health Survey of England (2006).

	Men		Women	
	Model 1	Model 2	Model 1	Model 2
**Hypertension**	Odds ratios		Odds ratios	
No risk	1 (ref)	1	1	1
Increased risk	1.69 (1.34 to 2.14)	1.67 (1.28 to 2.17)	1.31 (1.02 to 1.67)	1.20 (0.90 to 1.60)
High risk	1.95 (1.52 to 2.51)	1.73 (1.29 to 2.32)	2.00 (1.59 to 2.50)	1.79 (1.38 to 2.34)
Very high risk	2.57 (2.06 to 3.21)	2.54 (1.97 to 3.28)	2.15 (1.75 to 2.64)	2.18 (1.71 to 2.77)
P for trend	<0.001	<0.001	<0.001	<0.001
**Diabetes**	Odds ratios		Odds ratios	
No risk	1 (ref)	1	1	1
Increased risk	1.89 (1.11 to 3.21)	2.10 (1.06 to 4.17)	1.04 (0.47 to 2.27)	0.73 (0.24 to 2.25)
High risk	2.60 (1.53 to 4.43)	2.08 (1.00 to 4.32)	3.85 (2.25 to 6.59)	3.67 (1.89 to 7.10)
Very high risk	5.61 (3.59 to 8.76)	5.90 (3.28 to 10.64)	5.83 (3.61 to 9.42)	4.05 (2.18 to 7.55)
P for trend	<0.001	<0.001	<0.001	<0.001
**Raised total cholesterol**	Odds ratios		Odds ratios	
No risk	1 (ref)	1	1	1
Increased risk	1.51 (1.19 to 1.92)	1.58 (1.21 to 2.06)	1.31 (1.02 to 1.67)	1.25 (0.96 to 1.63)
High risk	1.34 (1.04 to 1.74)	1.63 (1.21 to 2.18)	1.39 (1.09 to 1.78)	1.60 (1.21 to 2.12)
Very high risk	1.18 (0.94 to 1.49)	1.28 (0.99 to 1.64)	1.36 (1.09 to 1.70)	1.50 (1.17 to 1.91)
P for trend	0.13	0.03	0.002	<0.001
**Low HDL**	Odds ratios		Odds ratios	
No risk	1 (ref)	1	1	1
Increased risk	0.69 (0.43 to 1.12)	0.55 (0.33 to 0.93)	1.39 (0.40 to 4.81)	1.23 (0.36 to 4.19)
High risk	0.40 (0.25 to 0.64)	0.36 (0.21 to 0.62)	0.77 (0.29 to 2.08)	0.69 (0.23 to 2.02)
Very high risk	0.33 (0.21 to 0.50)	0.26 (0.17 to 0.42)	0.49 (0.23 to 1.08)	0.72 (0.30 to 1.76)
P for trend	<0.001	<0.001	0.07	0.39
**HbA1C (mmol/l)**	β coefficients*		β coefficients*	
No risk	0 (ref)	0	0	0
Increased risk	0.13 (0.05 to 0.21)	0.13 (0.05 to 0.21)	0.05 (−0.00 to 1.00)	0.01 (−0.04 to 0.06)
High risk	0.32 (0.19 to 0.45)	0.26 (0.14 to 0.39)	0.23 (0.16 to 0.30)	0.15 (0.08 to 0.23)
Very high risk	0.40 (0.29 to 0.51)	0.38 (0.27 to 0.49)	0.39 (0.31 to 0.46)	0.31 (0.24 to 0.39)
P for trend	<0.001	<0.001	<0.001	<0.001
**Framingham risk score**	β coefficients*		β coefficients*	
No risk	0 (ref)	0	0	0
Increased risk	1.11 (0.73 to 1.49)	0.91 (0.64 to 1.19)	1.24 (0.71 to 1.76)	0.74 (0.34 to 1.14)
High risk	1.62 (1.20 to 2.05)	1.08 (0.75 to 1.41)	2.96 (2.43 to 3.49)	1.94 (1.52 to 2.36)
Very high risk	1.86 (1.52 to 2.21)	1.67 (1.40 to 1.94)	3.27 (2.80 to 3.75)	2.64 (2.25 to 3.03)
P for trend	<0.001	<0.001	<0.001	<0.001
**Fibrinogen (g/L)**	β coefficients*		β coefficients*	
No risk	0 (ref)	0	0	0
Increased risk	0.10 (0.01 to 0.19)	0.09 (0.01 to 0.18)	0.19 (0.10 to 0.27)	0.17 (0.08 to 0.25)
High risk	0.14 (0.04 to 0.24)	0.10 (0.01 to 0.20)	0.25 (0.16 to 0.33)	0.25 (0.16 to 0.33)
Very high risk	0.27 (0.18 to 0.37)	0.29 (0.19 to 0.39)	0.49 (0.41 to 0.56)	0.47 (0.39 to 0.55)
P for trend	<0.001	<0.001	<0.001	<0.001
**CRP (mg/L)**	β coefficients*		β coefficients*	
No risk	0 (ref)	0	0	0
Increased risk	1.43 (1.27 to 1.62)	1.45 (1.28 to 1.65)	1.56 (1.38 to 1.77)	1.58 (1.39 to 1.80)
High risk	1.82 (1.58 to 2.08)	1.77 (1.54 to 2.02)	2.01 (1.79 to 2.25)	2.01 (1.79 to 2.29)
Very high risk	2.16 (1.92 to 2.44)	2.17 (1.92 to 2.45)	3.60 (3.25 to 3.97)	3.49 (3.13 to 3.90)
P for trend	<0.001	<0.001	<0.001	<0.001

BMI: body mass index; WC: waist circumference; HDL: high density lipo-protein; HbA1C: glycated haemoglobin; CRP: C-reactive protein.

Model 1: unadjusted; Model 2: associations adjusted for age, social class, alcohol consumption, physical activity and smoking.

* Each regression coefficient (β) represents the amount of change in concentration of the biomarkers (logarithmic in case of CRP) per increase the risk category.

Similar results were obtained for women with the exception that total cholesterol levels were associated with the risk categories while HDL levels were not. Adjustments decrease the magnitude of the association with diabetes in women but still the dose-response along the risk categories was observed. When Framingham risk score was used as an outcome, as expected, similar results were obtained for men and women as were obtained for the risk factors which comprise this risk algorithm. Again, a gradient across the NICE adiposity categories was observed, with very high risk category found to have the highest coefficient of score. This trend was observed for men and women with much bigger beta coefficients for women than men.

## Discussion

Our main objective was to investigate the association of the NICE obesity guidelines for cardiovascular disease risk factors, and, in so doing, their predictive utility. Our results indicate that using the NICE categories of BMI and WC are useful in identifying people with increased risk of selected CVD risk factors. Using the NICE categories of obesity risk, there were less favourable levels of CVD risk factors in the ‘higher risk’ groups; these effects were generally linear.

### Comparison with Previous Studies

Our results essentially accord with other reports, mostly from the North American population [19], [30]–[32]. Using the National Institute of Health (NIH) clinical guidelines in the US population, Janssen et al [30] have shown that within the three BMI categories, those with higher WC values were likely to have unfavourable CVD outcomes compared with those with normal WC values; again, these effects were stepwise. Likewise, Arden et al. [31] have reported that the OR for the prediction of the metabolic syndrome were elevated in overweight and obese women but not men with a high WC compared with overweight and obese women with a low WC, respectively. Zhu et al [19] by using the National Health and Nutrition Examination Survey have shown that combined measures of BMI and WC may provide a higher overall test performance for CVD risk factors and may be useful in some ethnic groups as a means of screening subjects for further evaluation in the clinical setting. However, in a recent study of 58 cohorts from 17 countries, Wormser et al. [33] argued that BMI, waist circumference, and waist-to-hip ratio, whether assessed singly or in combination, do not importantly improve CVD risk prediction in people in developed countries when additional information is available for systolic blood pressure, history of diabetes, and lipids. The disagreement of these results might be due to the difference from race, age, study design, measurement method of WC, continuous or dichotomized variable for WC or using different categories of WC than what we have used in the current study.

### Mechanisms and Policy Implications

It is generally recognised that the central deposition of fat (abdominal or visceral obesity) is closely associated with chronic diseases and is a key constituent of the metabolic syndrome, a disorder characterised by increased risk of developing diabetes, stroke and cardiovascular disease [34]. Combined measures of BMI and WC can help identify more adults who might have elevations in CVD risk factors. Thus, results from our study have strengthened the fact that both BMI and WC are the screening CVD risk tools in England. Furthermore, the National Institutes of Health (NIH) [35] guidelines found that BMI gave a reasonable approximation of adiposity in most people and that waist circumference was the most practical measurement for assessing abdominal fat. The NICE evidence-based guidelines on obesity include details on prevention, identification, assessment and management of overweight and obesity, with one aim being to increase health professionals’ awareness of how to manage overweight and obesity in primary care. Our analyses have shown associations of overweight and obesity along with high or very high waist circumference on various risk factors of CVD. These results confirm the need for healthcare professionals to incorporate into clinical decision-making the NICE obesity guidelines which take into account both the waist circumference measurements and BMI. Treatment of overweight and obesity should be implemented through effective evidence-based weight management interventions such as those highlighted in the NICE guidelines, alongside broader preventive strategies at the population level.

### Strengths and Weaknesses of the Study

This study has several strengths. First, the findings were based on a large scale national level survey in England and this particular sample of HSE (2006) was especially designed to study cardiovascular disease risk in the population. By using the NICE obesity categories, we have validated the effectiveness of using the combined categories of BMI and WC in identifying the CVD risk factors. Additionally, we have shown that in the setting of England, the NICE obesity categories work more effectively than the combined categories of BMI and WC used in the US as recommended by NIH [35]. There are a number of limitations to this study which need to be considered It has been remained unclear and not very much debated that whether there should be a set of separate cut-offs for WC when used in combination with BMI [36]. This aspect needs further investigation. Any future research on this topic should address the associations of NICE obesity categories with mortality. Also, we did not address the issue of reverse causation, i.e. the possibility that CVD risk factors caused adiposity either independently or through other factors such as dietary intake and other life style factors. We did not have the data on other CVD risk factors such as IL6, triglycerides and low-density lipoprotein cholesterol. Finally, we were unable to do analysis stratified by race or ethnicity as the sample was 95% white.

### Conclusions

This is the first study in England which has demonstrated the effectiveness of the combined categories of BMI and WC in relation to CVD risk factors. Additionally, this study has used three cut-offs of WC instead of using a dichotomous WC allowing a more fine grained analysis. Our study suggests that CVD health risk is greater in overweight and obese for those who have high and very high WC compared with people with normal WC values.
